# The prevalence and genotype of 21-hydroxylase deficiency in the Croatian Romani population

**DOI:** 10.3389/fendo.2023.1170449

**Published:** 2023-05-31

**Authors:** Katja K. Dumic, Zorana Grubic, Vesna Kusec, Duje Braovac, Kristina Gotovac, Maja Vinkovic, Maja Vucinic, Miroslav Dumic

**Affiliations:** ^1^ Department of Paediatric Endocrinology and Diabetes, University Hospital Centre Zagreb, University of Zagreb School of Medicine, Zagreb, Croatia; ^2^ Tissue Typing Centre, Department of Transfusion Medicine and Transplantation Biology, University Hospital Centre Zagreb, University of Zagreb School of Medicine, Zagreb, Croatia; ^3^ Department of Laboratory Medicine, University Hospital Centre Zagreb, University of Zagreb School of Medicine, Zagreb, Croatia; ^4^ Department for Functional Genomics, Center for Translational and Clinical Research, University of Zagreb School of Medicine, University Hospital Center Zagreb, Zagreb, Croatia; ^5^ Department of Pediatrics, General Hospital Našice, Našice, Croatia

**Keywords:** congenital adrenal hyperplasia, founder mutation, Romani tribes, consanguinity, CYP21A2 heterozygous advantage, Romani Holocaust, bottle neck effect

## Abstract

**Objective:**

Congenital adrenal hyperplasia (CAH) owing to 21-hydroxylase deficiency (21-OHD) is a rare autosomal recessive disorder caused by pathological variants in the *CYP21A2* gene. After a high prevalence of classic 21-OHD CAH in the Romani population was reported in the Republic of North Macedonia, we decided to estimate the prevalence of 21-OHD in Croatia and, if high, assess the possible causes and estimate the frequency of particular *CYP21A2* variants.

**Design:**

Cross-sectional study.

**Methods:**

Data from a Croatian 21-OHD genetic database was reviewed, and only Romani patients were included in the study. *CYP21A2* genotyping was performed using allele-specific PCR, MLPA, and Sanger sequencing.

**Results:**

According to a survey conducted in 2017, Croatia had 22,500 Romani people and six of them had a salt-wasting (SW) form of 21-OHD. All were homozygous for the c.IVS2-13A/C-G pathological variant in intron 2 and descended from consanguineous families belonging to different Romani tribes. The calculated prevalence of 21-OHD in Croatian Romani is 1:3,750, while in the Croatian general population, it is 1:18,000. Three of the six Romani patients originated from two neighboring villages in North-western Croatia (Slavonia County), as well as the seventh patient who is of mixed Romani/Croatian descent and heterozygous for the c.IVS2-13A/C-G pathological variant (not included in the prevalence calculation).

**Conclusion:**

A high prevalence of SW 21-OHD in the Croatian Romani population caused by the homozygous cIVS2-13A/C-G pathological variant was found. In addition to isolation and consanguinity, other possible reasons could be the heterozygous advantage of the *CYP21A2* gene pathological variant and the bottleneck effect as a result of the Romani Holocaust in World War II.

## 1 Introduction

Congenital adrenal hyperplasia (CAH) due to 21-hydroxylase deficiency (21-OHD) caused by pathological variants in the *CYP21A2* gene has been classified into a) the classical form that includes the severe salt-wasting (SW) and milder simple-virilizing (SV) forms and b) the non-classical (NC) form. The range of disease manifestations, on the other hand, appears to be a spectrum with no defined boundaries (1). The intron 2 splicing variant c.IVS2-13A/C-G of the *CYP21A2* gene is the most prevalent variant causing 21-OHD, accounting for 25%–45% of all pathological variants in most major studies (2). The general incidence of the classical form of 21-OHD varies between 1:10,000 and 1:20,000 live births in different countries. However, the prevalence is substantially greater in some isolated ethnic groups such as the Alaskan Eskimos (1:250) and in the population of the island La Reunion, France (1:2,141) (3), as well as in highly consanguineous populations of Eastern Mediterranean (United Arab Emirates, Saudi Arabia, Kuwait, Alexandria-Egypt) with incidence larger than 1:10,000 (4).

Romani people who live in large numbers in European countries originated from northwestern India. Their migration began approximately in the 10th century (5), and various Romani groups were for the first time quoted in Croatia in the 14th century (6). After the abolition of slavery in Romania in 1855, the largest groups of Romani people migrated to Croatia in the 19th century (6).

During World War II, when more than 20,000 Croatian Romani were executed, the Romani population in the Independent State of Croatia plummeted (7).

According to the last Croatian census from 2011, there were 16,675 Romani living in Croatia (8). According to a survey conducted in 2017 by Ecorys Hrvatska d.o.o. and the Centre for Peace Studies NGO, there were approximately 22,500 Romani living throughout Croatia (9). The discrepancy between the census data and the estimated Romani population can be explained by the fact that many of them decided not to identify as Romani because of stigmatization, and thus, we believe that the 2017 survey data are more reliable. The Romani population in Croatia is divided into several tribal groups, the largest one being the Bayash, which accounts for more than 55% of the Croatian Romani. Other tribes such as Chergar, Kaloper, Lovar, Ashkalia, Gagar, and Kalderash are less numerous (approximately 10%–15% of Croatian Romani). However, more than 30% of the Romani people do not declare themselves or do not know to which tribe they belong (10).

Despite the government funding for various projects that assist Romani, they remain underserved, as they are in most other countries, and more than 90% of Croatia’s Romani population is at risk of poverty and dependent on social welfare. As a result of their isolation and endogamy, the Romani population does have a higher prevalence of several genetic disorders. However, the comprehensive report on genetic studies in the Romani population does not mention 21-OHD patients (5). Furthermore, in countries with a large Romani population, no Romani newborn with 21-OHD has been diagnosed through neonatal screening, but only in Slovakia ethnicity was recorded (11), while this was not the case in the programs implemented in Hungary (12) and the Czech Republic (13).

After Kocova et al. reported a high incidence of classic 21-OHD in the Romani population in the Republic of North Macedonia and found that 9/10 patients are homozygous for the c.IVS2-13A/C-G variant (14), we decided to estimate the prevalence and genotype of 21-OHD in Croatian Romani and, if high, assess the possible causes and estimate the frequency of particular *CYP21A2* pathological variants.

## 2 Patients and methods

*CYP21A2* gene analysis was performed in seven patients with 21-OHD (six Romani and one patient of mixed Romani/Croatian descent). All of them were followed at the Referral Centre for Paediatric Endocrinology, University Hospital Centre Zagreb, where *CYP21A2* gene analysis of all Croatian 21-OHD patients was performed and reported in a database. Ethnicity was assessed using parents’ or patient’s personal claim. Written informed consent for the publication of clinical details and results of molecular testing was obtained from the patients and their guardians.

Since a newborn screening program for 21-OHD in Croatia has not yet been introduced, diagnosis and disease classification were established by history, physical examination, electrolytes, and hormonal data. In both male and female 21-OHD patients, diagnosis of the SW form of the disease was based on failure to thrive, vomiting, dehydration, hyponatremia, hyperkalemia, elevated levels of 17-hydroxyprogesterone (17-OHP) and androstenedione, and increased plasma renin activity (PRA), and in female patients, the diagnosis was based on the appearance of ambiguous genitalia, classified by the Prader genital score (PGS). The criteria for the diagnosis of the SV form of the disease included ambiguous genitalia and pseudoprecocious puberty in female patients and increased height and bone age and elevated 17-OHP and androstenedione in both male and female patients.

### 2.1 Molecular genetic analysis

Firstly, allele-specific PCR for the eight most common pathological variants of the *CYP21A2* gene [p.P31L, c.IVS2-13A/C-G, p.Gly111Valfs*21, p.I173N, Ex6 cluster (p.I236N, p.V237E, p. M239K), p.V282L, p.Q319X, and p.R357W] was performed as described previously (15). Additionally, multiplex ligation-dependent probe amplification (MLPA) was used to detect deletions and large conversions (SALSA MLPA kit P050-B2, MRC Holland, Amsterdam, Netherlands), and in order to detect rare variants, Sanger sequencing was performed (15, 16).

The study was approved by the Ethics Committee of the Department of Paediatrics, University Hospital Centre Zagreb, University of Zagreb Medical School, Croatia.

## 3 Results

The study included six SW 21-OHD patients of Romani descent, one boy and five girls (one reared as a boy), as well as one boy of mixed Romani/Croatian descent. All patients were diagnosed as newborns.

Considering the survey conducted in 2017 that approximately 22,500 Romani lived in 15 counties in Croatia, the calculated prevalence of 21-OHD in this population is 1:3,750 (of note, patient #7 who is of Romani/Croatian descent is not included in the calculation). In contrast, during the same period, 4,160,357 non-Romani residents lived in Croatia, with 221 classical 21-OHD non-Romani individuals reported in our database, giving the predicted prevalence of 1:18,825 (15).

The clinical characteristics and laboratory data of the six Romani patients and one patient of Romani/Croatian descent with CAH due to 21-OHD are presented in **Table 1**.

**Table 1:** Clinical and hormonal data of the six Romani patients and one patient of Romani/Croatian descent with congenital adrenal hyperplasia due to 21-hydroxylase deficiency.

Family	A		B	C	D	E	F
Patient no.	1	2	3	4	5	6	7
Sex	F	M	F	M	F	F	M
Declared ethnicity	Kalderash		Bayashi	Bayashi	Bayashi	Ashkalia	Not declared
Consanguinity	+	+	+	+	+	+	−
Age at diagnosis	15 days	13 days	16 days	32 days	15 days	18 days	19 days
17-OHP (n.v. 0.9–12.1 nmol/L)	162	174	153	233	211	189	321
Androstenedione (n.v. 1–8 nmol/L)	14.2	15.2	12.6	16.4	15.4	13.7	15.1
PRA (n.v. 2.8–16.1 μg/L/h)	19.8	20.6	23.4	22.6	24.1	18.8	29.83

After being diagnosed with SW 21-OHD in the neonatal period and early infancy, all patients were started on treatment with hydrocortisone, fludrocortisone, and salt. With the exception of patients #6 and #7, in the other five patients, therapy was taken irregularly and they rarely came to scheduled visits. Surgical correction of the genitalia was performed in patients #3, #5, and #6 between the ages of 2 and 4.

Patients #1 and #2 were siblings whose parents were related, and they were declared as Romani Kalderash. The family was from a small Romani population settled in the suburbs of the city of Split in Dalmatia County. Patient #1 was born with ambiguous genitalia (PGS IV), karyotype was 46, XX, but the patient was declared and brought up as a male at the request of the parents, and surgical correction of genitalia was not performed. At the age of 6 months, he died at home of an adrenal crisis. His younger brother died at the age of 3 weeks of unknown causes. Another boy (patient #2) was born afterward and was diagnosed with 21-OHD SW CAH.

Four patients lived in the small geographic area in North-western Croatia (Slavonia County), and three of them (#3, #4, and #5) declared themselves as Bayash Romani. The fourth patient (#7), who was of mixed Romani/Croatian descent, did not declare further regarding ethnicity. Patients #3 and #4 who were related were from a small village of Podgorac, where according to the 2017 survey there were 866 inhabitants, 8% of which were Romani. The other two (#5 and #7) were from the neighboring village of Vukojevci, with 992 inhabitants (according to the 2017 survey), 33% of which were Romani. Furthermore, we found out that the father of patient #7 from Vukojevci was related to the father of patient #3 from Podgorac as well as to the mother of patient #5 from Vukojevci.

Patient #6 is a daughter of Ashkalia Romani (Albanized Romani) parents who were related and migrated from Kosovo to Croatia. Ashkalia Romani represent only approximately 3% of all declared Romani in Croatia, and most of them are settled in the Istra/Primorje County where patient #6 is living.

**Figure 1:**
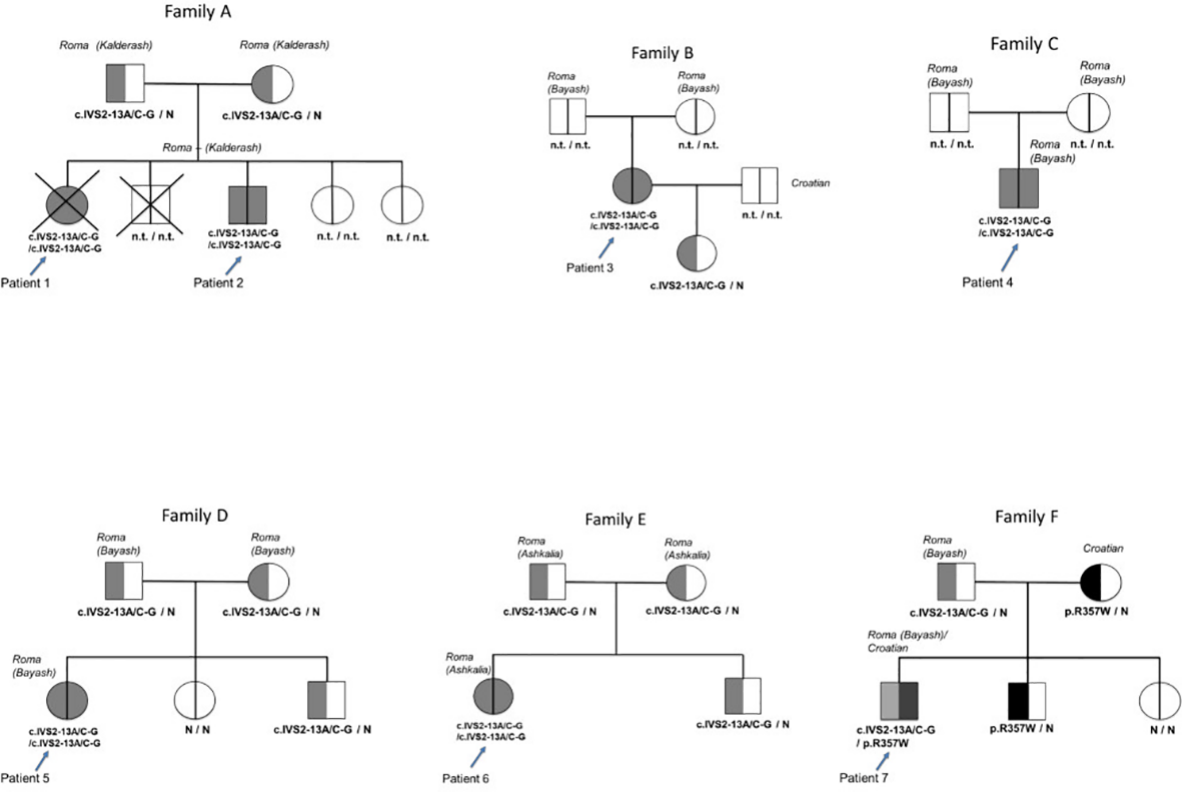
Pedigrees and genotypes of the six Romani patients and one mixed Romani/Croatian patient with classical congenital adrenal hyperplasia due to 21-OH deficiency. N.t., not tested; N, pathological variant in the *CYP21A2* gene not found.

The *CYP21A2* gene variant analysis was performed in all seven patients and 14 family members [parents of patients #1, #2, #5, #6, and #7; siblings of patients #5, #6, and #7; and daughter of patient #2 (in total 42 alleles)] (**Figure 1**).

Patients #1 to #6 were homozygous for the c.IVS2-13A/C-G pathological variant. Patient #7 was a compound heterozygote with the c.IVS2-13A/C-G pathological variant inherited from the father who was of Bayash Romani origin and the p.R357W pathological variant inherited from the mother who was of Croatian origin. Heterozygosity for the c.IVS2-13A/C-G variant was detected in the parents of patients #1, #2, #5, and #6, the brother of patient #5, the brother of patient #6, and the daughter of patient #3. Moreover, the sisters of patient #5 and the brother of patient #7 were healthy homozygotes (**Figure 1**).

## 4 Discussion

Approximately 10 million Romani scattered in European countries are usually described as a conglomerate of the genetically isolated founder population. The Romani populations are generally underserved and have very low socioeconomic status and education level, as well as limited access to healthcare. Therefore, it is hard to conduct an epidemiological study that would offer trustworthy data on the incidence/prevalence of specific diseases among the Romani population. Bartsocas et al. studied the genetic structure of Greek Romani and concluded that there is no evidence of hereditary diseases except color blindness. However, the family history in this study revealed relatives with SW 21-OHD (17). Furthermore, some genetic diseases, such as inborn errors of metabolism (phenylketonuria, galactosemia, medium-chain and short-chain acyl-CoA dehydrogenase deficiency, and carnitine uptake defect), autosomal dominant polycystic kidney disease, and primary congenital glaucoma, have been shown to be more common in Romani compared with the other populations, in part due to increased use of molecular genetic diagnostics (5). Similar clustering of rare diseases and private founder variants has been observed in other founder populations, including Jews, Finns, and French Canadians (5), as well as the Yupik Inuit of Western Alaska, who have a high incidence of SW 21-OHD (1:282) and exclusive homozygosity for the c.IVS2-13A/C-G pathological variant of the *CYP21A2* gene (3, 18).

Kocova et al. recently demonstrated that 21-OHD should be considered as one of the common genetic diseases in the Romani population. In the Republic of North Macedonia, they found a high incidence of 21-OHD (1:3,375) in the Romani community, while the estimated incidence in other ethnicities was 1:14,000. They also found that 9/10 Romani patients had severe SW CAH and all of them had the c.IVS2-13A/C-G/c.IVS2-13A/C-G genotype, whereas the genotype of the other ethnic groups in the Republic of North Macedonia was variable. They attributed their patients’ high homozygosity for the c.IVS2-13A/C-G pathological variant to frequent endogamy due to isolation and low admixture with surrounding ethnicities. Moreover, they speculated that 21-OHD is more common in North Macedonia Romani or that patients’ ethnicity was not adequately recorded in other European countries with large Romani populations (12, 13). The exception, however, is Slovakia with approximately five million inhabitants predominantly of European origin and a significant minority of 500,000 Romani, where follow-up ethnicity has been introduced in an extended screening program. During 3 years of screening (from 2013 to 2015), a total of 165,648 newborns had been tested, of which 25,321 (15.4%) were Romani and 140,327 (84.7%) were of European origin (11). There were 10 newborns detected with 21-OHD (1:16,565), all of whom were European and none of whom were Romani, which is surprising given the high incidence and prevalence of 21-OHD among North Macedonian and Croatian Romani (9). Both in North Macedonia, where a high incidence of 21-OHD was found in the Romani population, and in Slovakia, where no patients of Romani descent were found, the exact tribal affiliation was not declared (11, 14). Given the fact that the Romani population is heterogeneous, it would be very interesting to know tribal affiliation, because it could help us explain 21-OHD incidence differences in the Romani population in various countries.

It is assumed that the prevalence of the classical form of 21-OHD in Croatia, both in the general population and among the Romani, is higher than estimated. In the general population, this is primarily because some of the patients with 21-OHD, especially those of older age, were not registered. Regarding Romani, it is to be expected that some patients are not kept in the registry, as well as some have never been diagnosed and/or died, especially those who were born at home.

Regarding the earlier investigation of the classical form of 21-OHD in the Croatian population, one larger population study has been carried out so far, where we reported an incidence of 1:14,403 for classical 21-OHD in the period between 1995 and 2006 (19). The ethnicity of the patients was not recorded.

In our study of the prevalence of 21-OHD in Croatian Romani, we also had difficulty estimating the total number of Romani people in Croatia, as well as the number of patients with 21-OHD among them, due to their unwillingness to cooperate and the unreliability of the data they provided. However, considering the data from the survey conducted in 2017, which estimated that approximately 22,500 Romani live in Croatia, the results of our study indicated a very high prevalence of 1:3,750 of classical 21-OHD in the Croatian Romani population. In contrast, the prevalence of classical 21-OHD in the Croatian population is estimated to be 1:18,825.

The high prevalence of classical 21-OHD in the Romani population can be explained by their seclusion, infrequent admixture with other ethnicities, and consanguinity, as seen by the fact that all six patients are homozygous for the c.IVS2-13A/C-G pathological variant. The only compound heterozygote, patient #7, inherited his c.IVS2-13A/C-G variant from his Romani father and his p.R357W variant from his Croatian mother. He presented with the salt-wasting phenotype. The fact that four out of seven of our patients with related parents (#3, #4, #5, and #7) were from two neighboring villages in Slavonia County with a total population of approximately 350 Romani and that patients #1, #2, #5, and #6 were also from consanguineous families but lived in different parts of Croatia confirms that variants circulate within the same families.

In 2017, we conducted a molecular genetic investigation of 93 Croatian patients with conventional 21-OHD and discovered that 34.9% of them had the c.IVS2-13A/C-G pathological variant. Eighteen out of 90 patients (19.3%) were homozygous for the c.IVS2-13A/C-G variant (16 SW and two SV) (15). As in the study on the incidence of classical 21-OHD in Croatia (19), the ethnicity of the patients was not recorded, and further review of the records revealed that two Romani (#5 and # 6) and one patient of mixed origin (#7) were included in both studies (15, 19).

Our findings are consistent with those of Kocova et al., who hypothesized that the c.IVS2-13A/C-G pathological variant is a founder variant originating from an ancestral variant that has been preserved due to isolation of the Romani people, infrequent mixing with other ethnic groups, and endogamy (14).

Generally, the c.IVS2-13A/C-G pathological variant represents the most frequent variant of the *CYP21A2* gene associated with the classical 21-OHD, primarily with the SW form of the disease. However, it has been shown that the carriers of c.IVS2-13A/C-G either in homozygous or compound heterozygous status in combination with other pathological variants of the *CYP21A2* gene can present with different and variable phenotypes. Interestingly, even intrafamilial phenotypic discordance (SW and SV as well as the NC form) can be observed in patients with identical homozygous genotypes for c.IVS2-13A/C-G (20).

Regarding the Croatian and North Macedonian Romani population, all patients homozygous for the c.IVS2-13A/C-G pathological variant presented with the SW form of 21-OHD, and intrafamilial phenotype discordance has not been found.

Interestingly, one should also mention that although in most autosomal recessive traits homozygotes for the mutant allele have reduced fitness compared with heterozygotes or homozygous for the normal allele, there are some disorders where the fitness of heterozygotes is greater than that for both homozygous types. This heterozygote advantage phenomenon has been reported in carriers of severe *CYP21A2* pathological variants who have defined phenotype with a prompter cortisol response to ACTH compared with healthy homozygous carriers (21). This increased capacity to synthesize cortisol in acute situations could explain their overall lower mortality when compared with healthy homozygous carriers. Several studies support this finding showing that carriers tolerate stress better and have reduced risk of psychiatric diagnosis, death due to infection (especially severe pneumonia), and risk for metabolic syndrome development and atherogenesis (22–24). Since a small heterozygote advantage will increase the frequency of the mutant allele in the population even in carriers of severe diseases like CAH, it is not a surprise that the carrier frequency of *CYP21A2* is high. The heterozygous advantage such as this one certainly increases carrier frequency to some degree in populations like Romani that live in a low socioeconomic environment with limited access to medical care and are more exposed to infectious diseases. Another important factor can be the bottleneck effect. Before World War II, the number of Romani in Croatia was approximately 27,000–28,000. During the World War II Romani Holocaust—Projamos, more than 20,000 Romani in Croatia were executed, while others mostly migrated, which led to the virtual extinction of this population in Croatia. According to the first post-war Croatian census conducted in 1948 (8), only 405 Romani were evidenced. Subsequently, the original gene pull was reduced, and a high birth rate of Croatian Romani which is 4.5 times higher than in the general population (45% *vs*. 9.8%) (25) could lead to low genetic variability in this fast-expanding population.

In conclusion, a high prevalence of SW 21-OHD CAH in the Croatian Romani population due to the homozygous c.IVS2-13A/C-G pathological variant in the *CYP21A2* gene was discovered. In addition to endogamy and low admixture rate with other ethnic groups, we assume that the possible reasons for such high prevalence could be the heterozygous advantage of the *CYP21A2* gene variant and the bottleneck effect. It would be interesting to investigate the prevalence/incidence of classical 21-OHD and perform genotyping in other nations with a large Romani population, recording their tribal affiliation.

## Data availability statement

The original contributions presented in the study are included in the article/supplementary material, further inquiries can be directed to the corresponding author/s.

## Ethics statement

The studies involving human participants were reviewed and approved by Ethics Committee of the Department of Paediatrics, University Hospital Centre Zagreb, University of Zagreb Medical School, Croatia. The patients/participants provided their written informed consent to participate in this study.

## Author contributions

KDK, ZG and MD: conceptualization, methodology, and writing—review and editing. MVI, DB, MVU, KG and VK: resources, data collection, and analysis. KDK: original draft preparation. MD and ZG: supervision. All authors contributed to the article and approved the submitted version.
